# Single-specificity anti-Ku antibodies in an international cohort of 2140 systemic sclerosis subjects: clinical associations

**DOI:** 10.1097/MD.0000000000004713

**Published:** 2016-09-02

**Authors:** S. Hoa, M. Hudson, Y. Troyanov, S. Proudman, J. Walker, W. Stevens, M. Nikpour, S. Assassi, M.D. Mayes, M. Wang, M. Baron, M.J. Fritzler, Canadian Scleroderma Research Group (CSRG); Australian Scleroderma Interest Group (ASIG); Genetics versus Environment in Scleroderma Outcome Study (GENISOS)

**Affiliations:** a Department of Medicine, McGill University, Montreal, Quebec, Canada; b Lady Davis Institute, Jewish General Hospital, Montreal, Quebec, Canada; c Division of Rheumatology, Jewish General Hospital, Montreal, Quebec, Canada; d Division of Rheumatology, Hopital du Sacre-Coeur de Montreal, Montreal, Quebec, Canada; e Department of Medicine, Université de Montréal, Montreal, Quebec, Canada; f Rheumatology Unit, Royal Adelaide Hospital, Adelaide, Australia; g Discipline of Medicine, University of Adelaide, Bedford Park, Australia; h Department of Allergy and Immunology, Flinders Medical Centre, Bedford Park, Australia; i Department of Rheumatology, St. Vincent's Hospital Melbourne, Fitzroy, Victoria, Australia; j Department of Medicine, The University of Melbourne at St. Vincent's Hospital, Melbourne, Victoria, Australia; k Division of Rheumatology and Immunogenetics, University of Texas Health Science Centre at Houston, Houston, TX; l Faculty of Medicine, University of Calgary, Calgary, Alberta, Canada.

**Keywords:** anti-Ku antibodies, international cohort, interstitial lung disease, single-specificity, systemic sclerosis

## Abstract

Autoantibodies directed against the Ku autoantigen are present in systemic sclerosis (SSc) and have been associated with myositis overlap and interstitial lung disease (ILD). However, there is a paucity of data on the clinical correlates of anti-Ku antibodies in the absence of other SSc-specific antibodies. The aim of this study was to assess the clinical correlates of single-specificity anti-Ku in SSc.

An international (Canada, Australia, USA, Mexico) cohort of 2140 SSc subjects was formed, demographic and clinical variables were harmonized, and sera were tested for anti-Ku using a line immunoassay. Associations between single-specificity anti-Ku antibodies (i.e., in isolation of other SSc-specific antibodies) and outcomes of interest, including myositis, ILD, and survival, were investigated.

Twenty-four (1.1%) subjects had antibodies against Ku, and 13 (0.6%) had single-specificity anti-Ku antibodies. Subjects with single-specificity anti-Ku antibodies were more likely to have ILD (58% vs 34%), and to have increased creatine kinase levels (>3× normal) at baseline (11% vs 1%) and during follow-up (10% vs 2%). No difference in survival was noted in subjects with and without single-specificity anti-Ku antibodies.

This is the largest cohort to date focusing on the prevalence and disease characteristics of single-specificity anti-Ku antibodies in subjects with SSc. These results need to be interpreted with caution in light of the small sample. International collaboration is key to understanding the clinical correlates of uncommon serological profiles in SSc.

## Abstract 2

Supplemental Digital Content is available in the text

## 1 Introduction

Systemic sclerosis (SSc) is a heterogeneous disease with varying degrees of skin and organ involvement, and can be classified by extent of skin involvement (limited or diffuse cutaneous SSc), and also by serological subtype. Common SSc-specific autoantibodies, such as anticentromere (ACA), antitopoisomerase I (ATA), and anti-RNA polymerase III (ARNAP) antibodies, have been associated with specific clinical features. In recent years, less common SSc-associated autoantibodies have been studied and their clinical correlates characterized. A potential limitation of some of those studies is the confounding introduced by the presence of overlapping antibodies. The study of distinct autoantibodies in the absence of other SSc-related autoantibodies, which we will refer to as single-specificity, has allowed us to understand specific clinical correlates of individual autoantibodies. For example, Ro52/TRIM21 autoantibodies were found to be independently associated with the presence of interstitial lung disease (ILD) and poor survival in SSc,^[1]^ and distinct associations were found for single-specificity anti-PM75, anti-PM100, and anti-PM-1α antibodies.^[2,3]^

Autoantibodies directed against Ku have been reported in a small percentage of SSc sera. The Ku (p70/p80) antigen is a DNA-binding protein involved in doubled-stranded DNA repair, through the nonhomologous end-joining pathway.^[4–8]^ It combines with a DNA-dependent protein kinase catalytic subunit (DNA-PKcs) and regulates the phosphorylation of many nuclear proteins, including nuclear enzymes and transcription factors.^[9]^ It also plays a role in V(D)J recombination of receptor genes on B and T lymphocytes,^[4–8]^ immunoglobulin class switching,^[10]^ telomere protection,^[11]^ and development of the central nervous system.^[12]^

The prevalence of anti-Ku autoantibodies in SSc varies from 1.5% to 16%,^[13–25]^ depending primarily on the detection immunoassay, and on the genetic and geographical background of the subjects studied.^[26]^ They were first described in 1981 by Mimori et al^[19]^ as a marker of scleroderma-polymyositis overlap syndrome, but have since been reported in a variety of other autoimmune disorders, including systemic lupus erythematosus (SLE) (0.7%–27%), idiopathic inflammatory myopathies (up to 26%), mixed connective tissue disease and undifferentiated connective tissue disease (up to 8.3%), rheumatoid arthritis (up to 16%), and Sjögren syndrome (<1%–20%), in isolation or as part of overlap syndromes,^[13,14,19,23–25,27–47]^ and only rarely in healthy controls.^[19,23,25]^ In SSc, these autoantibodies have been associated with myositis^[14,17,19,22,32,42,48]^ and ILD,^[14,42]^ and also limited cutaneous involvement,^[14,19,22]^ arthritis,^[14,22]^ and less vascular involvement.^[14,20–22]^ However, results have been conflicting,^[13,22,24,25]^ and conclusions have been limited by small numbers of subjects studied and potentially confounded by the co-presence of other SSc-related autoantibodies.

The objective of this study was therefore to identify the demographic, clinical, and serological characteristics of SSc subjects with single-specificity anti-Ku antibodies in a large international, multicenter cohort.

## 2 Methods

An international (Canada, Australia, USA, Mexico) retrospective cohort of 2140 SSc subjects was formed, demographic and clinical variables were harmonized, and sera were tested for anti-Ku using a line immunoassay (LIA). Associations between single-specificity anti-Ku antibodies (i.e., in isolation of other SSc-related antibodies), baseline characteristics, and mortality were investigated.

### 2.1 Sources of data

The study subjects were SSc patients enrolled in the Canadian Scleroderma Research Group (CSRG), the Australian Scleroderma Interest Group (ASIG), or the American Genetics versus Environment in Scleroderma Outcome Study (GENISOS) cohorts. Briefly, subjects in the CSRG are recruited from 15 sites across Canada and Mexico, and must have a diagnosis of SSc verified by an experienced rheumatologist, be >18 years of age, and be fluent in English, French, or Spanish. Over 98% of the cohort meets the 2013 American College of Rheumatology (ACR)/European League Against Rheumatism (EULAR) classification criteria for SSc.^[49]^ Loss to follow-up in the CSRG cohort is 25%. Subjects in the ASIG are recruited by investigators from 12 Australian centers specializing in the care of patients with SSc, according to similar inclusion criteria. All subjects fulfill either the 1980 preliminary ACR criteria for classification of SSc, or the Medsger criteria for limited SSc.^[50]^ Estimated loss to follow-up in the ASIG cohort is 7%. The GENISOS cohort is a longitudinal cohort of subjects with early SSc. Subjects are enrolled within 5 years of disease onset as determined by the first non-Raynaud phenomenon symptom from 3 University of Texas institutions at Houston, San Antonio, and Galveston. All enrolled subjects fulfill the 2013 ACR/EULAR classification criteria for SSc.^[51]^ Estimated loss to follow-up in the GENISOS cohort is 25%.

Ethics committee approval for this study was obtained at McGill University (Montreal, Canada) and at all participating CSRG, ASIG, and GENISOS study sites. All subjects provided informed written consent to participate in the study. The subjects included in this study were those whose baseline visits were between September 2004 and June 2014 for CSRG, between January 2007 and March 2013 for ASIG, and between January 1998 and September 2012 for GENISOS, and who had complete serological profiles for anti-Ku antibodies as detected by the methods described below.

### 2.2 Clinical variables

Subjects recruited into this study underwent standardized medical evaluation including medical histories, physical examinations and laboratory investigations, according to the protocols from their respective cohorts, and the following clinical variables were harmonized to create a single dataset with common variable definitions. All study variables were collected at the baseline study visit, except creatine kinase (CK) and mortality, which were also available during follow-up.

Demographic information regarding age, sex, and ethnicity was collected by patient self-report. Disease duration was recorded by study physicians and defined as the interval between the onset of the first non-Raynaud disease manifestation and baseline study visit.

Skin involvement was assessed using the modified Rodnan Skin Score (mRSS), a widely used clinical assessment where the examining rheumatologist records the degree of skin thickening ranging from 0 (no involvement) to 3 (severe thickening) in 17 areas (total score range 0–51). Limited cutaneous disease was defined as skin involvement distal to the elbows and knees with or without facial involvement; diffuse cutaneous disease was defined as skin involvement proximal to the elbows and knees with or without truncal involvement. Those with a clinical diagnosis of SSc but no skin involvement were included with the limited cutaneous subset.

History of inflammatory myositis, calcinosis, inflammatory arthritis, scleroderma renal crisis, and malignancy was recorded by a study physician. The presence of telangiectasias, digital pits, and digital ulcers on physical examination was also recorded by a study physician. CK levels were measured by local laboratories.

To assess gastrointestinal involvement, subjects answered yes/no to 6 questions concerning gastroesophageal reflux disease, dysphagia, antibiotics for bacterial overgrowth, episodes of pseudo-obstruction, fecal incontinence, and hyperalimentation.

The presence of ILD was determined using a clinical decision rule that was recently published.^[52]^ This algorithm considers ILD to be present if a high-resolution computed tomography (HRCT) scan of the lung was interpreted by an experienced radiologist as showing ILD, or, in the case where no HRCT is available, if either a chest x-ray was reported as showing increased interstitial markings (not thought to be due to congestive heart failure) or fibrosis, and/or if a study physician reported the presence of typical “velcro-like crackles” on physical examination. Pulmonary function tests were performed at local respiratory physiology laboratories.

Pulmonary hypertension was defined as an estimated systolic pulmonary artery pressure (sPAP) ≥45 mm Hg measured using the Doppler flow measurement of the tricuspid regurgitant jet on cardiac echocardiography (an estimate that correlates strongly with right heart catheter studies)^[53]^ for CSRG and GENISOS subjects, or mean pulmonary artery pressure (mPAP) >25 mm Hg with a pulmonary capillary wedge pressure (PCWP) <15 mm Hg on right heart catheterization for ASIG subjects.

In addition, disease overlap with SLE and Sjögren syndrome, history of Raynaud phenomenon, trigeminal neuralgia, and autoimmune thyroid disease, and presence of capillaroscopic alterations on dermatoscopic examination recorded by a study physician were also available in the CSRG dataset.

All subjects were assessed, followed, and classified in the same way, on a similar platform, regardless of anti-Ku antibody status and outcomes. Frequency of missing data was recorded.

### 2.3 Serology

Autoantibody analysis of the CSRG and GENISOS cohorts were performed in a central laboratory—Mitogen Advanced Diagnostics Laboratory, University of Calgary—and the ASIG analyses were performed using an identical immunoassay kit and protocol. Serum aliquots were stored at −80°C until needed for diagnostic assays. Antinuclear antibodies (ANAs) were detected by indirect immunofluorescence (IIF) performed on HEp-2 cells (ImmunoConcepts, Sacramento, CA). Anti-Ku, centromere (CENP-A and CENP-B), topoisomerase I, RNA polymerase III (RP11 and RP155), fibrillarin, NOR-90, Th/To, Ro52/TRIM21, PDGFR, PM75, and PM100 antibodies were detected by Euroline SSc profile LIA (Euroimmun GmbH, Luebeck, Germany) according to manufacturer's instructions. With the intent of optimizing specificity, antibodies were reported as absent (negative, equivocal, and low titers) and present (moderate and high titers). Data on ANA titers and patterns were also available for subjects from the CSRG cohort.

### 2.4 Statistical analysis

Subjects were grouped according to anti-Ku status, either positive (further subdivided into single-specificity or overlapping with other SSc antibodies) or negative at baseline visit. Descriptive statistics were used to summarize the baseline demographic and clinical characteristics of the subjects. Given the exploratory nature of the analysis and the small samples in the subgroups, clinically relevant numerical differences between subgroups were considered informative. Exploratory statistical analyses were performed using chi-square tests, Fisher exact tests, and Mann–Whitney *U* tests, as indicated. *P* < 0.05 was considered statistically significant. Bonferroni correction for multiple testing was calculated for statistically significant findings. Missing data, selection bias, and information bias were addressed qualitatively.

Kaplan–Meier analysis and Cox proportional-hazard models adjusting for baseline differences in age, ethnicity, and sex were used to compare survival between autoantibody subsets. Multivariate logistic regression adjusting for baseline differences in age and ethnicity was used to determine the association between anti-Ku antibody groups and ILD. *P* values <0.05 were considered statistically significant.

All statistical analyses were performed with SAS v.9.2 (SAS Institute, Cary, NC).

## 3 Results

All cohort subjects were tested for anti-Ku antibodies and were eligible for inclusion. Of the 2140 SSc subjects included in this study, 24 (1.1%) had anti-Ku antibodies. Thirteen (0.6%) had single-specificity anti-Ku antibodies (i.e., in isolation of other SSc-related antibodies), 11 (0.5%) had overlapping anti-Ku antibodies, and 2116 (98.9%) were negative for anti-Ku antibodies (Table 1). Individual clinical and serological characteristics of single-specificity and overlapping anti-Ku-positive subjects are presented in Tables 2 and 3, respectively.

**Table 1:**
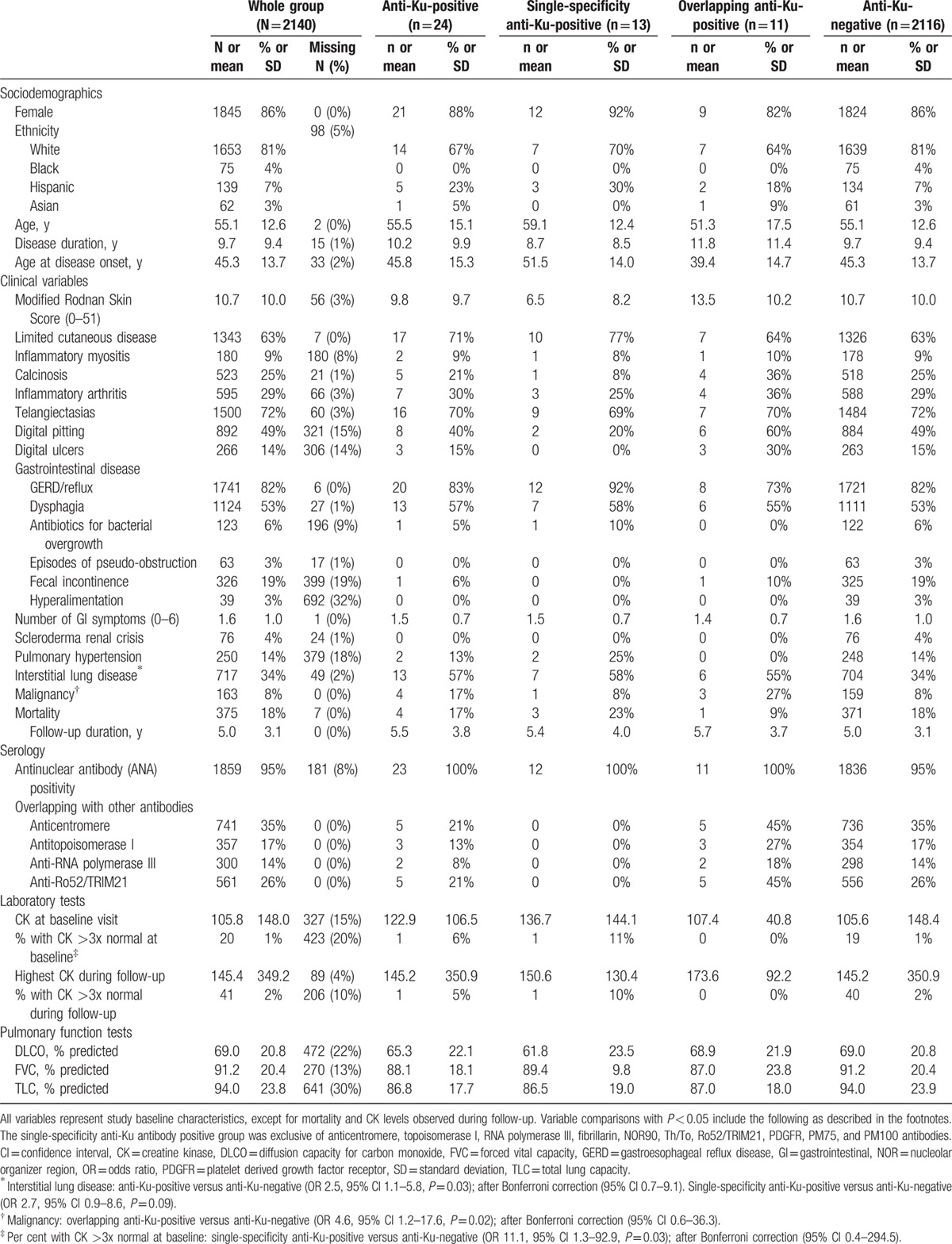
Baseline characteristics of the study cohort, as a group and according to anti-Ku antibody status.

**Table 2:**
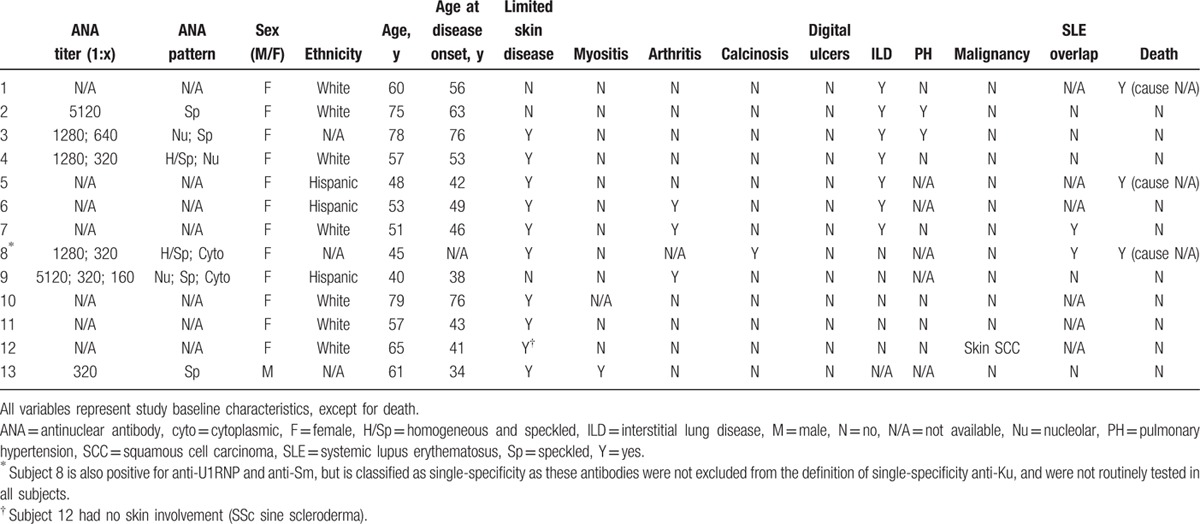
Clinical and serological characteristics of single-specificity anti-Ku-positive subjects.

**Table 3:**
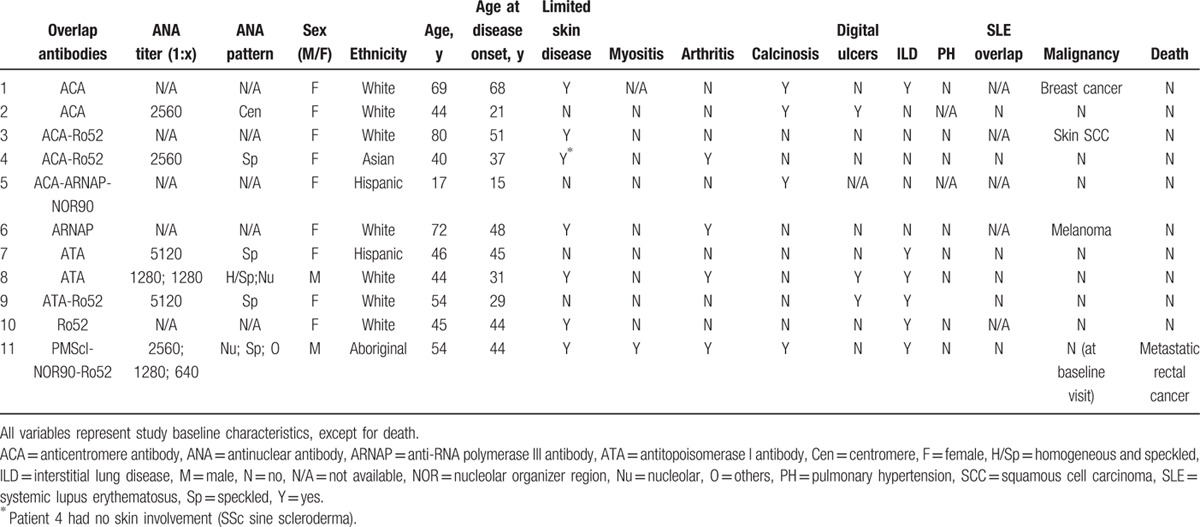
Clinical and serological characteristics of overlapping anti-Ku-positive subjects.

### 3.1 Clinical correlates of single-specificity anti-Ku-positive subjects

Subjects with single-specificity anti-Ku antibodies tended to be older at disease onset (mean age 51.5 vs 45.3 years), of Hispanic ethnicity (30% vs 7%), and with limited cutaneous disease (77% vs 63%); and less likely to be of white ethnicity (70% vs 81%), have digital pitting (20% vs 49%), digital ulcers (0% vs 15%), and calcinosis (8% vs 25%), compared with anti-Ku-negative subjects.

Interstitial lung disease was also more common in single-specificity anti-Ku-positive subjects than in anti-Ku-negative subjects (58% vs 34%; odds ratio [OR] 2.7, 95% confidence interval [CI] 0.9–8.6, *P* = 0.09; in logistic regression analysis adjusting for differences in baseline demographic characteristics: OR 2.69, 95% CI 0.75–9.59, *P* = 0.13) (Tables 1 and 4).

**Table 4:**
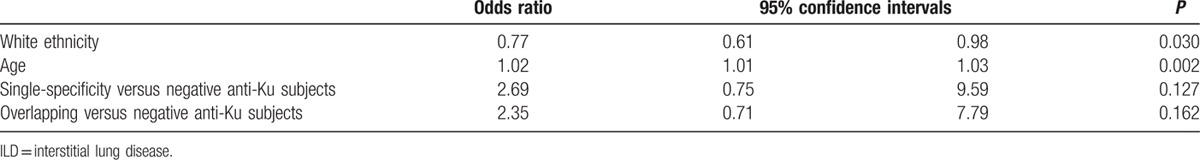
Multivariate logistic model to estimate the association between the presence of anti-Ku antibodies and ILD, adjusting for baseline demographic differences.

Pulmonary hypertension was numerically more common in single-specificity anti-Ku-positive subjects compared with anti-Ku-negative subjects (25% vs 14%; OR 2.0, 95% CI 0.4–10.0, *P* = 0.39).

Although there was no difference in inflammatory myositis prevalence (8% vs 9%), subjects with single-specificity anti-Ku antibodies were more likely to have significantly elevated CK levels (>3× normal) at baseline (11% vs 1%; OR 11.1, 95% CI 1.3–92.9, *P* = 0.03) and during follow-up (10% vs 2%).

Inflammatory arthritis was not more frequent in anti-Ku-positive subjects.

In a survival analysis adjusted for differences in baseline characteristics, subjects with single-specificity anti-Ku antibodies were not found to be at significantly increased risk of death compared with subjects without anti-Ku antibodies (mean [SD] follow-up of 5.0 [3.1] years) (Table 5 and Supplementary Figure).

**Table 5:**
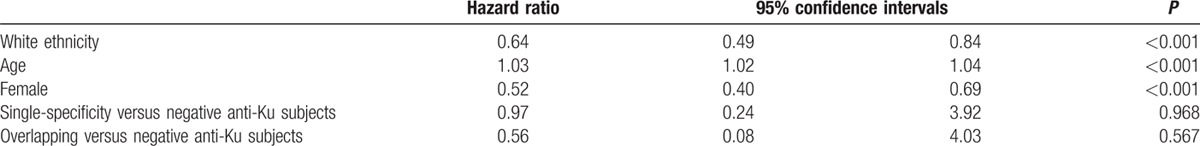
Cox proportional-hazard model to estimate the association between the presence of anti-Ku antibodies and mortality, adjusting for baseline demographic differences.

### 3.2 Exploratory findings in anti-Ku-positive subjects

Interestingly, subjects with overlapping anti-Ku antibodies were more likely to have a history of malignancy at baseline visit compared with anti-Ku-negative subjects (27% vs 8%,; OR 4.6, 95% CI 1.2–17.6, *P* = 0.03). The subjects with overlapping anti-Ku antibodies and malignancy had melanoma (ARNAP overlap), breast cancer (ACA overlap), and squamous cell skin cancer (ACA and anti-Ro52/TRIM21 overlap), respectively, none of which occurred within 2 years of SSc diagnosis. In comparison, the frequency of malignancy in single-specificity anti-Ku, ARNAP, and ACA-positive SSc subjects were 8.0%, 7.7%, and 8.9%, respectively.

In the CSRG cohort (comprising 7 single-specificity anti-Ku-positive and 1323 anti-Ku-negative subjects), overlap disease with SLE (28.6% vs 3.3%; OR 11.6, 95% CI 2.2–61.6, *P* = 0.004) was reported more frequently in single-specificity anti-Ku subjects compared with anti-Ku-negative subjects. Frequency of Sjögren syndrome (0% vs 7.4%), trigeminal neuralgia (0% vs 2.6%), autoimmune thyroid disease (0% vs 12.3%), Raynaud phenomenon (85.7% vs 97.4%), or abnormal capillaroscopy (85.7% vs 76.5%) was not significantly different in single-specificity anti-Ku-positive subjects compared with anti-Ku-negative subjects (Supplementary Table 1).

### 3.3 Serological characteristics of anti-Ku-positive subjects

All subjects with anti-Ku antibodies had positive ANA by IIF (Table 1). In the CSRG cohort, all subjects with single-specificity anti-Ku antibodies (n = 6) had ANA titers of at least 1:320 with speckled patterns, along with nucleolar patterns in half of subjects and cytoplasmic patterns in a third of subjects (Table 2 and Supplementary Table 2).

Among subjects with overlapping antibodies, 6 had 1 more (2 ACA, 2 ATA, 1 ARNAP, 1 Ro52/TRIM21), 3 had 2 more (2 ACA-Ro52/TRIM21, 1 ATA-Ro52/TRIM21), and 2 had 3 more (1 ACA-RNAP-Nor90, 1 PM-Scl-Nor90-Ro52/TRIM21) overlapping antibodies. Interestingly, the majority of subjects who had ILD had either ATA or anti-Ro52/TRIM21 overlapping antibodies, whereas most subjects with calcinosis had ACA overlapping antibodies (Table 3).

Of note, 1 CSRG subject classified as part of the single-specificity anti-Ku-positive group also had positive anti-U1-RNP and anti-Sm autoantibodies (which were not among the antibodies tested for the whole sample, and therefore not excluded from the definition of single-specificity anti-Ku-positivity in this study). This subject was diagnosed with SSc-SLE overlap disease and was the only single-specificity anti-Ku-positive subject who had calcinosis (Table 2).

## 4 Discussion

We aimed to describe the demographic, clinical, and serological characteristics of SSc subjects with single-specificity anti-Ku antibodies. In this international, multicenter cohort of 2140 subjects, only 24 (1.1%) had anti-Ku antibodies, and 13 (0.6%) had single-specificity anti-Ku antibodies. These numbers are slightly lower than previously reported frequencies using the LIA technique (Table 6) and might be attributed to our intent of optimizing specificity by using a higher cut-off (moderate and high titers only). Single-specificity anti-Ku-positive subjects in this cohort tended to be older, have more limited skin disease, and less vascular digital complications than anti-Ku-negative subjects. ILD was more frequent in anti-Ku-positive subjects in general, and also in single-specificity and overlapping anti-Ku-positive subjects. CK elevations were also more common in subjects with single-specificity anti-Ku antibodies. Serologically, single-specificity anti-Ku-positive subjects had high-titer speckled ANAs, with or without nucleolar staining patterns.

**Table 6:**
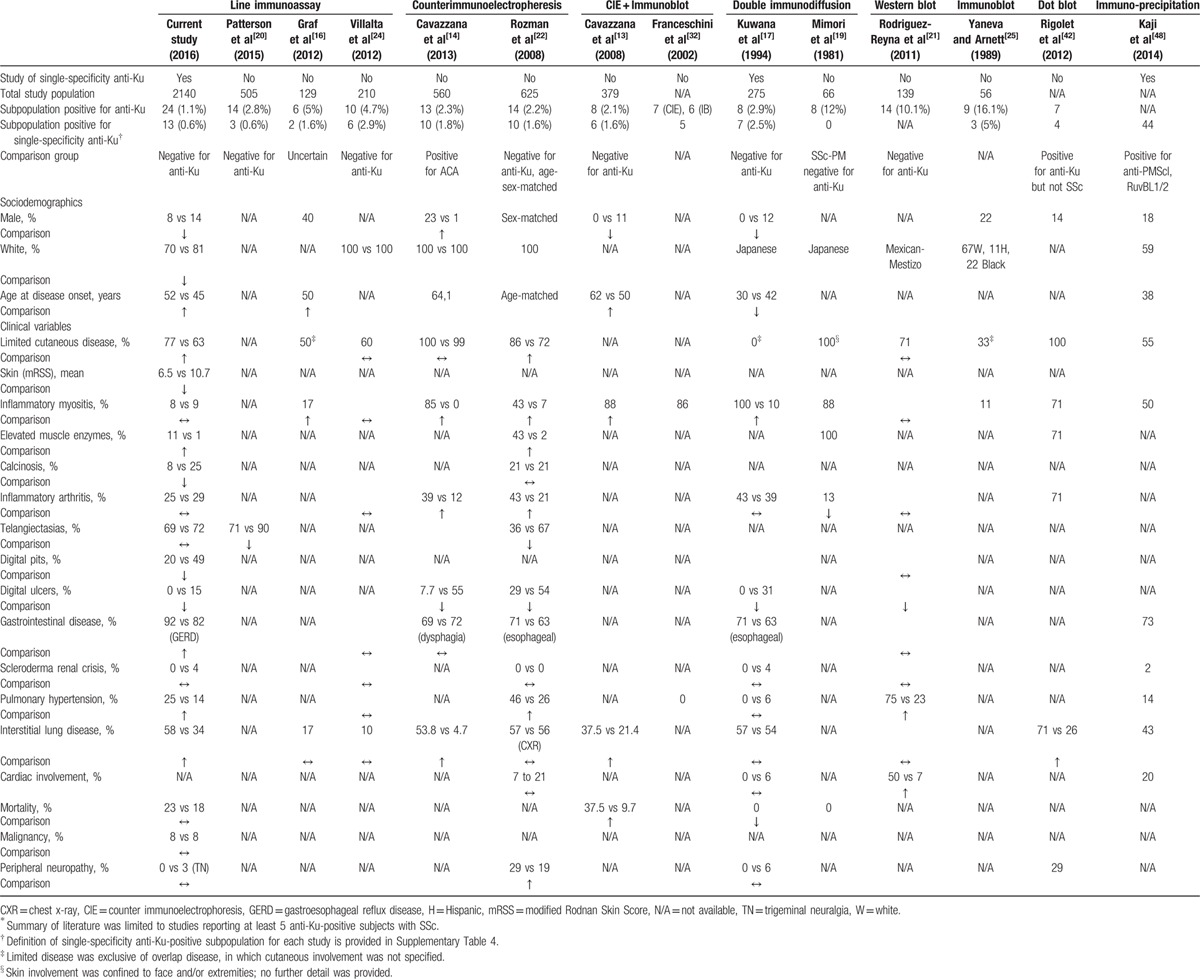
Summary of the literature on clinical associations of anti-Ku antibodies in SSc, by method of detection.^∗^

To date, little was known on the clinical correlates of single-specificity anti-Ku antibodies in SSc. In an extensive review of the literature (Table 6, Supplementary Table 4), only 2 studies were identified that examined these antibodies in isolation. The first, by Kuwana et al,^[17]^ reported on 7 Japanese subjects with SSc and single-specificity anti-Ku antibodies; in contrast to our study, they were found to have a younger age of disease onset compared with anti-Ku-negative subjects. All were classified as overlap SSc syndromes, all were associated with skeletal muscle involvement, and none had digital tip ischemia. The second study, by Kaji et al,^[48]^ compared 44 SSc subjects (from 2 Japanese institutions and the University of Pittsburgh) with single-specificity anti-Ku antibodies with anti-RuvBL1/2 and anti-PM-Scl-positive subjects, all considered as related to SSc/myositis overlap; 50% had inflammatory myositis and 43% had ILD. However, no comparison with triple-negative subjects was provided. Three additional studies from European centers^[13,14,22]^ reported on anti-Ku-positive SSc subjects in whom the majority (>70%) had single-specificity anti-Ku antibodies. Subjects were found to be older at disease onset,^[13]^ have more limited cutaneous involvement,^[14,22]^ inflammatory myositis,^[13,14,22]^ inflammatory arthritis,^[14,22]^ and trigeminal neuralgia,^[22]^ and less digital vascular complications.^[14,22]^ ILD was also more frequent, but was characterized by mild functional impairment.^[13,14]^

On the contrary, lung disease associated with anti-Ku-positive myositis with or without SSc overlap has been reported to be corticosteroid refractory in 75% of subjects by Rigolet et al.^[42]^ Furthermore, studies of anti-Ku-positive inflammatory myopathy subjects in which at least half had single-specificity autoantibodies have also shown an association with more ILD,^[42,44]^ inflammatory arthritis,^[36,44]^ overlap with other rheumatic diseases,^[36,44]^ and milder inflammatory myopathies, as evidenced by less frequent dermatomyositis rash,^[31,45]^ modest CK elevations,^[31,42]^ nonspecific abnormalities on muscle biopsy,^[31]^ and treatment-responsive, monophasic course of muscle disease.^[42,54]^

Our findings are generally consistent with previously reported clinical associations between anti-Ku autoantibodies in SSc and limited cutaneous involvement, ILD, and less vascular complications, and strengthen these findings by showing an association with single-specificity anti-Ku autoantibodies in a large multicenter patient sample.

Pulmonary hypertension has been previously reported to be associated with anti-Ku antibodies. Rodriguez-Reyna et al^[21]^ found that 73% of anti-Ku-positive SSc subjects had pulmonary arterial hypertension, compared with only 23% of anti-Ku-negative SSc subjects. We also found a higher rate of pulmonary hypertension among the single-specificity anti-Ku subjects (25%), although this observation was based on only 2 subjects.

Other features previously associated with anti-Ku autoantibodies, such as higher rates of myositis overlap, were not clearly observed in our cohort: only 8% of single-specificity anti-Ku-positive subjects had inflammatory myositis, which was similar to other subgroups. However, CK elevations were more common in the single-specificity anti-Ku subjects, although this observation was based on only 1 subject. Interestingly, most of the studies that reported very high rates of myositis overlap (71%–90%) identified anti-Ku-positive subjects through screening of sera positive for autoantibodies to extractable nuclear antigens (ENAs).^[13,14,19,32,42,55]^ On the contrary, studies that analyzed anti-Ku-positive subjects by screening a SSc population with a LIA technique such as in our study, did not report such a high prevalence of myositis.^[16,24]^ Furthermore, Cooley et al^[29]^ previously observed that anti-Ku-positive subjects who met classification criteria for a connective tissue disease tended to meet the minimum number of criteria. This was again demonstrated in the study by Hausmanowa-Petrusewicz et al,^[36]^ in which 5 anti-Ku-positive subjects had sclerodactyly and telangiectasias, but only 2 were identified as scleromyositis or SSc-polymyositis overlap, the 2 others remaining “unclassified,” given that their muscle and SSc-spectrum diseases did not the meet classification criteria. As such, it is possible that a number of subjects with anti-Ku-positivity and a diagnosis of inflammatory myositis may present milder clinical features of SSc, such as sclerodactyly, puffy fingers, Raynaud phenomenon, or esophageal dysmotility, which are all suggestive of an overlap disease with SSc, but in isolation may not be classified as SSc disease. Therefore, these subjects may not be referred and captured into a cohort of SSc, which could explain the lower frequency of myositis in association with anti-Ku antibodies in SSc cohort studies.

Interestingly, SSc-SLE disease overlap was more frequent in single-specificity anti-Ku-positive subjects in our cohort; anti-Ku antibodies in SLE subjects have not previously been associated with a particular clinical phenotype, except for African ethnicity.^[13,14,25,33]^ On the contrary, trigeminal neuralgia and autoimmune thyroid disease were not seen in anti-Ku-positive subjects, contrary to what has been observed with anti-Ku in a few case reports and series.^[22,42,56–58]^

Of note, clinical characteristics of single-specificity and overlapping subjects tended to be distinct on many levels: age, cutaneous extent, frequency of digital vascular complications, calcinosis and malignancy, and mortality. This dataset highlights the importance of studying single-specificity autoantibodies, as overlapping subjects may present a different, likely mixed phenotype.

All international subjects with anti-Ku antibodies had ANA by IIF, and all CSRG subjects with single-specificity anti-Ku autoantibodies had titers of at least 1:320, all of speckled with or without nucleolar staining patterns (Table 2). This is consistent with anti-Ku's serological characteristics previously reported in the literature (Supplementary Table 3). We also observed that anti-Ku-positive subjects generally had less frequent concomitant ACA (21% vs 35%); this is consistent with findings by Rozman et al,^[22]^ who found decreased concomitant ACA and ATA autoantibodies in anti-Ku-positive subjects. On the contrary, Graf et al^[16]^ found increased association between anti-Ku and antifibrillarin (or U3RNP) autoantibodies, whereas none of our anti-Ku-positive subjects had this autoantibody (Supplementary Table 3). It is acknowledged, however, that the LIA used in our international cohort has a low sensitivity as compared with immunoprecipitation and other immunoassays (M.J. Fritzler, unpublished data, April 2016).

The role of anti-Ku in the pathophysiology of autoimmune diseases is not entirely understood. The autoantibody-binding target Ku is known to be involved in double-stranded DNA repair.^[4–8]^ Schild-Poulter et al^[23]^ found that anti-Ku autoantibodies were often associated with autoantibodies directed against other DNA repair proteins, and suggested that B-cell responses to latent or persistent DNA damage may be involved at the onset or during the development of autoimmunity in certain systemic autoimmune rheumatic diseases. Hypoxia has also been reported to induce chromatin modifications, leading to recruitment and activation of the DNA-dependent protein kinase (which includes Ku)^[59]^; this is of interest given that vasculopathy and tissue hypoxia are thought to be part of the initial pathophysiology of SSc. Ku is also involved in V(D)J recombination of receptor genes on B and T lymphocytes^[4–8]^ and in immunoglobulin class switching^[10]^; one could hypothesize that defective expression of the Ku peptide may lead to altered function of the immune system and result in autoimmunity as well. Genetic background seems to play a role in anti-Ku autoimmunity, as evidenced by its positive association with certain human leukocyte antigen (HLA) class II genotypes.^[55,60]^ Molecular mimicry between the Ku antigen and certain fungal proteins has also been postulated as a potential trigger for anti-Ku autoimmunity in genetically predisposed individuals.^[61]^

Finally, overlapping anti-Ku antibodies were found to be associated with a history of malignancy in our study. To our knowledge, this association has not been reported previously (Table 6). Interestingly, malignancy has been hypothesized to act as a trigger of autoimmunity in certain cases of SSc, particularly in the ARNAP-positive subset, via mechanisms involving antitumor immunity, molecular mimicry, and epitope spreading.^[62,63]^ In fact, tumors associated with ARNAP-positive SSc have been shown to harbor mutated forms of the RNA polymerase III autoantigen.^[64]^ In the same way, genetic alterations of the Ku antigen in tumor cells could explain the association between malignancy and overlapping anti-Ku antibodies in SSc. Alternatively, overexpression of repair proteins (such as Ku) in response to DNA damage intrinsic to cancer cells, or defective expression of Ku leading to both uncontrolled tumor expansion and immune system dysfunction, constitute other hypotheses to link anti-Ku autoimmunity and malignancy.

This study has some limitations. Inflammatory myositis was not defined using specific criteria. Instead, a study physician reported its presence or absence. However, the fact that all study physicians were experienced rheumatologists supports the validity of this diagnosis. Nevertheless, mild myositis may have been overlooked. Similarly, defining ILD in the context of longitudinal observational cohort studies is very complex, given issues of missing data and verification bias. We defined ILD using a clinical decision rule that was recently published.^[52]^ Data on right heart catheterization was not systematically collected in all subjects. Nevertheless, in those without right heart catheterization, we defined pulmonary hypertension using a high cut-off for pulmonary systolic pressure on echocardiogram that has been shown to correlate strongly with right heart catheter studies.^[53]^ Still, we acknowledge that pulmonary hypertension based on echocardiogram is not synonymous with pulmonary arterial hypertension, and that some of those with pulmonary hypertension based on echocardiogram may have had other causes of pulmonary hypertension such as left heart disease or parenchymal lung disease. Thus, measurement error may have contributed to some of the negative findings of the study.

In addition, the LIA used in this study to detect anti-Ku does not distinguish reactivity to the p70 and/or p80 subunits. In an international cohort study of 73 anti-Ku-positive subjects with different connective tissue diseases, 21 of whom had SSc, Lakota et al^[39]^ found a positive association between females with anti-Ku-p70 and joint/bone features (defined as synovitis, joint contractions, erosive arthritis, and acroosteolysis), but a negative association between females with anti-Ku-p80 and joint/bone features. Furthermore, in SSc, 38% had isolated anti-Ku-p70, 10% had isolated anti-Ku-p80, and 43% had both.^[39]^ Yaneva and Arnett^[25]^ also found that anti-Ku-p86 levels were highest at the onset of disease and decreased and plateaued over the following years, whereas anti-Ku-p70 antibody levels remained fairly constant. The clinical phenotype described in our study could thus still represent a mixture of 2 clinical phenotypes. Further stratifying by reactivity to the 2 subunits could lead to the identification of a “purer” phenotype associated with anti-Ku subunits, similar to the findings that anti-PM-75 and anti-PM-100 are associated with distinct phenotypes.^[2]^ However, the rarity of these antibodies poses an enormous challenge to further stratification.

Furthermore, subjects identified as having “single-specificity” anti-Ku antibodies may in fact have had other autoantibodies that were not detected by the LIA employed in this study. This might include some associated with connective tissue disease-related ILD such as anti-Jo1, or markers of overlapping disease such as U1RNP, which were only available for a subset of the cohort. However, in the CSRG cohort, we have previously reported a very low prevalence of anti-Jo1 antibodies (approximately 1%),^[65]^ and previous studies have not reported any association between anti-Ku and anti-Jo1 or other antisynthetase antibodies in SSc.^[14,22,42]^ As for U1RNP, 1 case of overlapping Ku and U1RNP autoantibodies out of 13 anti-Ku-positive subjects (8%) was detected in the CSRG cohort, which is similar to the 7% to 13% frequency reported elsewhere.^[17,19,22]^ This subject was classified as having single-specificity anti-Ku antibodies, and was reported to have calcinosis, SLE overlap, and a fatal outcome (cause unknown). If this subject were to be removed from analyses, the single-specificity anti-Ku-positive group would display absence of calcinosis, increased frequency of SLE (17% vs 3%), and similar mortality (15% vs 18%) as anti-Ku-negative subjects, which is no different from current conclusions. Thus, the presence of these autoantibodies is unlikely to have influenced the results of this study in a meaningful manner.

Additional limitations in cohort studies include missing data (>10% for certain variables, such as pulmonary hypertension, CK levels, and pulmonary function test results) and loss to follow-up. However, given that data collection and follow-up were performed on a similar platform irrespective of anti-Ku status, missing data and loss to follow-up could be considered to be missing completely at random. Also, this cohort was composed predominantly of ambulatory patients with mean disease duration of 9.7 years. Thus, it lacks some generalizability for patients with early-onset disease, for those who may have died earlier in the course of their disease, and for seriously ill patients requiring hospitalization. Nevertheless, about a third of the cohort had disease duration of 5 years or less, and the whole cohort is representative of the majority of SSc patients seen in clinical practice. A final limitation of this study is the fact that most reported associations did not reach statistical significance. Given the exploratory nature of the analysis and the small samples in the subgroups, clinically relevant numerical differences were considered informative. Still, it remains possible that some of our findings occurred by chance alone.

On the contrary, when dealing with uncommon serological profiles (there were only 0.6% of subjects with single-specificity anti-Ku antibodies), large well-phenotyped cohorts are required to begin to fill important gaps in knowledge. In the end, the limitations of our data are counter-balanced by its strengths, which include large sample size and detailed clinical phenotypic data.

In conclusion, this is the largest cohort to date focusing on the prevalence and disease characteristics of single-specificity anti-Ku antibodies in subjects with SSc. In our international cohort, anti-Ku antibodies were rare, being found in only 1.1% of subjects. Nevertheless, as a clinician, if faced with a SSc patient who presents a milder cutaneous, vascular and possibly muscular disease phenotype, and who has strongly positive speckled ANA with or without nucleolar pattern, but has an otherwise negative panel for other tested SSc-specific antibodies anti-Ku could be suspected and tested for. If positive, increased clinical vigilance for ILD screening may be warranted. On the other hand, the usually mild clinical features and lack of survival difference associated with this autoantibody would be reassuring in terms of prognosis. Due to the very rare presence of these antibodies and thus the small size of the single-specificity anti-Ku sample, these results need to be interpreted with caution. International collaborations are key to understanding the clinical correlates of uncommon serological profiles in SSc.

## Supplementary Material

Supplemental Digital Content
