# Supplementary material for: Single-specificity anti-Ku antibodies in an international cohort of 2140 systemic sclerosis subjects: clinical associations
Source: Medicine (Baltimore). 2016 Sep 2;95(35):e4713. doi: 10.1097/MD.0000000000004713 (PMC5008592; doi:10.1097/MD.0000000000004713)
Supplement: Supplemental Digital Content [file medi-95-e4713-s001.doc]

**Supplementary Figure 1. Kaplan Meier curve comparing survival in anti-Ku single-specificity, overlapping and negative subjects.**


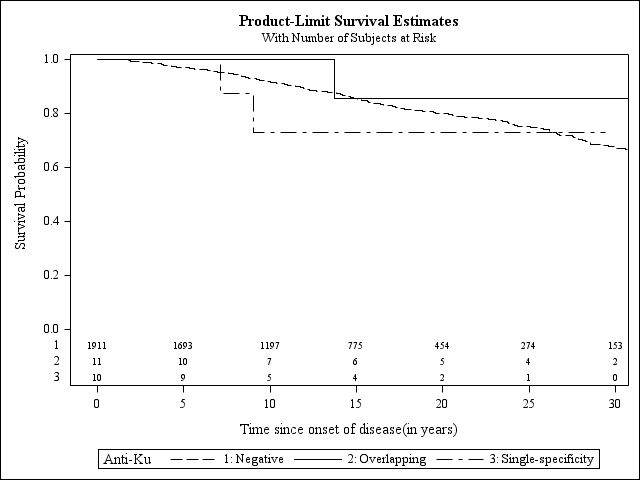


Subjects with single-specificity anti-Ku antibodies were not found to be at significantly increased risk of death compared to subjects without anti-Ku antibodies. Log rank p values: Single-specificity vs. negative 0.5920; overlapping vs. negative 0.3998.

**Supplementary Table 1. Baseline characteristics of the CSRG study cohort, as a group and according to anti-Ku antibody status.** The single-specificity anti-Ku antibody positive group was exclusive of anti-centromere, topoisomerase I, RNA polymerase III, fibrillarin, NOR90, Th/To, Ro52, PDGFR, PM75 and PM100 antibodies.

|  | Whole group (N=1336) | | | Anti-Ku positive  (N=13) | | Single-specificity anti-Ku positive (N=7) | | Overlapping anti-Ku positive (N=6) | | Anti-Ku negative (N=1323) | |
| --- | --- | --- | --- | --- | --- | --- | --- | --- | --- | --- | --- |
|  | N | % | N (%) Missing | N | % | N | % | N | % | N | % |
| SLE overlap* | 45 | 3.5% | 31 (2%) | 2 | 15.4% | 2 | 28.6% | 0 | 0% | 43 | 3.3% |
| Sjogren syndrome | 96 | 7.4% | 31 (2%) | 1 | 7.7% | 0 | 0% | 1 | 16.7% | 95 | 7.4% |
| Autoimmune thyroid disease | 32 | 12.2% | 1073 (80%) | 0 | 0% | 0 | 0% | 0 | 0% | 32 | 12.3% |
| Trigeminal neuralgia | 34 | 2.6% | 10 (1%) | 0 | 0% | 0 | 0% | 0 | 0% | 34 | 2.6% |
| Abnormal capillaroscopy | 1017 | 76.6% | 8 (1%) | 11 | 84.6% | 6 | 85.7% | 5 | 83.3% | 1006 | 76.5% |
| Raynaud's phenomenon | 1294 | 97.4% | 7 (1%) | 12 | 92.3% | 6 | 85.7% | 6 | 100% | 1282 | 97.4% |
| Meet 2013 ACR-EULAR classification criteria | 1296 | 97.6% | 8 (1%) | 12 | 92.3% | 6 | 85.7% | 6 | 100% | 1284 | 97.6% |

Abbreviations: CSRG – Canadian Scleroderma Research Group; NOR – nucleolar organizer; PDGFR – platelet derived growth factor receptor;.SLE – systemic lupus erythematosus. * SLE overlap: Single-specificity anti-Ku positive vs. anti-Ku negative, OR 11.6, 95% CI 2.2-61.6, p=0.0039; after Bonferroni correction, 95% CI 1.2-114.6.

Supplementary Table 2. Anti-nuclear antibody characteristics of the CSRG cohort, as a group and according to anti-Ku antibody status

|  | Whole group (N=1104) | | Anti-Ku positive (N=12) | | Single-specificity anti-Ku positive (N=6) | | Overlapping anti-Ku positive (N=6) | | Anti-Ku negative (N=1092) | |
| --- | --- | --- | --- | --- | --- | --- | --- | --- | --- | --- |
|  | N | % | N | % | N | % | N | % | N | % |
| *Titers of ANA* |  |  |  |  |  |  |  |  |  |  |
| 1:160 | 325 | 29% | 1* | 8% | 1* | 17% | 0 | 0% | 324 | 30% |
| 1:320 | 243 | 22% | 4* | 33% | 4* | 67% | 0 | 0% | 239 | 22% |
| 1:640 | 378 | 34% | 2 | 17% | 1 | 17% | 1 | 17% | 376 | 34% |
| 1:1280 | 433 | 39% | 6 | 50% | 3 | 50% | 3 | 50% | 427 | 39% |
| 1:2560 | 443 | 40% | 3 | 25% | 0 | 0% | 3 | 50% | 440 | 40% |
| 1:5120 | 219 | 20% | 4 | 33% | 2 | 33% | 2 | 33% | 217 | 20% |
| *Patterns of ANA* |  |  |  |  |  |  |  |  |  |  |
| Homogeneous | 36 | 3% | 0 | 0% | 0 | 0% | 0 | 0% | 32 | 3% |
| Homogeneous & speckled | 196 | 18% | 3 | 25% | 2 | 33% | 1 | 17% | 186 | 18% |
| Speckled | 540 | 49% | 8 | 66% | 4 | 66% | 4 | 66% | 514 | 49% |
| Nucleolar | 496 | 45% | 5 | 42% | 3 | 50% | 2 | 33% | 449 | 43% |
| Centromere | 400 | 36% | 1 | 8% | 0 | 0% | 1 | 17% | 397 | 38% |
| Cytoplasmic | 59 | 5% | 1 | 8% | 1 | 17% | 0 | 0% | 78 | 7% |
| Cytoplasmic speckled | 106 | 10% | 1 | 8% | 1 | 17% | 0 | 0% | 99 | 9% |
| Others | 208 | 19% | 1 | 8% | 0 | 0% | 1 | 17% | 168 | 16% |

Abbreviations: ANA – anti-nuclear antibody; CSRG – Canadian Scleroderma Research Group. Subjects may have more than one ANA titer and pattern. *Includes one cytoplasmic fluorescence titer.

**Supplementary Table 3.** Summary of the literature on serological characteristics of anti-Ku antibodies in SSc

|  | Line immunoassay | | | |  | CIE + LIA |  | Counterimmunoelectrophoresis | | |  | Counterimmunoelectro-phoresis + Immunoblot | |  | Double immunodiffusion | |  | Western blot |  | Immunoblot | |  | Dot blot |
| --- | --- | --- | --- | --- | --- | --- | --- | --- | --- | --- | --- | --- | --- | --- | --- | --- | --- | --- | --- | --- | --- | --- | --- |
|  | Current study (2016) | Patterson [20], 2015 | Graf [16], 2012 | Villalta [24], 2012 |  | Lakota [39], 2012 |  | Cavazzana [14], 2013 | Rozman [22], 2008 | Cooley [29], 1999 |  | Cavazzana [13], 2008 | Francescini [32], 2002 |  | Kuwana [17], 1994 | Mimori et [19], 1981 |  | Rodriguez-Reyna [21], 2011 |  | Yaneva [25], 1989 | |  | Rigolet [42], 2012 |
|  |  |  |  |  |  |  |  |  |  |  |  |  |  |  |  |  |  |  |  |  |  |  |  |
| ***Anti-nuclear antibody (ANA)*** |  | N/A | N/A | N/A |  |  |  |  |  |  |  |  |  |  | N/A |  |  |  |  |  |  |  |  |
| Study population: SSc or CTD | SSc |  |  |  |  | CTD |  | CTD | SSc | CTD |  | CTD | CTD |  |  | SSc |  | SSc |  | CTD | CTD |  | CTD |
| ANA of single-specificity anti-Ku | Yes |  |  |  |  | No |  | Yes | No | No |  | No | No |  |  | No |  | No |  | No | Yes |  | No |
| Number of subjects studied | 5 |  |  |  |  | 73 |  | 24 | 14 | 27 |  | 30 | 14 |  |  | 9 |  | 14 |  | 26 | 9 |  | 34 |
| Positivity, % | 100 |  |  |  |  | 99 |  | N/A | 100 | 100 |  | 100 | 100 |  |  | 89 (liver) |  | 100 |  | 92 | 78 |  | 100 |
| Titres | ≥1:640 |  |  |  |  | N/A |  | N/A | N/A | ≥1:640 |  | N/A | ≥10240 |  |  | ≥1:1024 |  | N/A |  | N/A | N/A |  | ≥1:320 |
| Pattern: speckled, % | 66 |  |  |  |  | Mostly |  | 71?, dense |  | 19 |  | 50, fine | 21 |  |  |  |  | N/A |  | 63 | 71 |  | Very dense speckled |
| Pattern: speckled + nucleolar, % | 50 |  |  |  |  | Mostly |  | 29 |  | 74 |  | 50, homog. nucleolar | 79 |  |  |  |  | N/A |  | 4 | 14 |  | Homog. nucleolar |
| Pattern: others | H/Sp 33 Cyto 17 |  |  |  |  |  |  |  | Nu 86 vs. 26 | H/Sp 4 H 4 |  |  |  |  |  | Reticular, sparing nucleoli |  |  |  | Cen 17 H 13 Spindle 4 | Spindle 14 |  | Cyto mitotic cell: diffuse, sharp-edged staining |
|  |  |  |  |  |  |  |  |  |  |  |  |  |  |  |  |  |  |  |  |  |  |  |  |
| ***Associated overlapping antibodies in anti-Ku positive SSc subpopulation*** | | | | |  |  |  | N/A |  |  |  | N/A |  |  |  |  |  | N/A |  |  |  |  |  |
| Number of SSc positive for anti-Ku | 24 | 14 | 6 | 10 |  | N/A |  |  | 14 | 4 |  |  | 7 |  | 8 | 8 |  |  |  | 9 | |  | 7 |
| Comparison group | Negative for anti-Ku | Negative for anti-Ku |  |  |  |  |  |  | Negative for anti-Ku, age-sex-matched |  |  |  |  |  |  |  |  |  |  |  |  |  |  |
| ACA, % | 21 vs. 35 | 36 | 50 | 10 |  | 0 |  |  | 0 vs. 35 |  |  |  |  |  | 0 |  |  |  |  | 44 | |  | 14 |
| Comparison | 🡻 | 🡸🡺 |  |  |  |  |  |  | 🡻 |  |  |  |  |  |  |  |  |  |  |  |  |  |  |
| ATA, % | 13 vs. 17 | 7 | 0 | 20 |  |  |  |  | 7 vs. 21 | 25 |  |  |  |  | 0 |  |  |  |  | 11 | |  | 0 |
| Comparison |  | 🡸🡺 |  |  |  |  |  |  | 🡻 |  |  |  |  |  |  |  |  |  |  |  |  |  |  |
| ARNAP, % | 8 vs. 14 | 21 | 17 | 10 |  |  |  |  |  |  |  |  |  |  | 0 |  |  |  |  |  |  |  |  |
| Comparison |  | 🡸🡺 |  |  |  |  |  |  |  |  |  |  |  |  |  |  |  |  |  |  |  |  |  |
| Ro52, % | 21 vs. 26 | 36 | N/A | N/A |  |  |  |  | 14 (Ro/La) | 25 (Ro) |  |  | 29 (Ro60) |  |  | 44 (Ro) |  |  |  |  |  |  |  |
| Comparison |  | 🡸🡺 |  |  |  |  |  |  |  |  |  |  |  |  |  |  |  |  |  |  |  |  |  |
| U3RNP (fibrillarin), % | 0 vs. 1 | 21 vs. 1 | 0 | 0 |  |  |  |  |  |  |  |  |  |  | 0 |  |  |  |  |  |  |  |  |
| Comparison |  | 🡹 |  |  |  |  |  |  |  |  |  |  |  |  |  |  |  |  |  |  |  |  |  |
| U1RNP, % | 8 (CSRG) | N/A | 0 | N/A |  |  |  |  | 7 |  |  |  |  |  | 13 | 11 |  |  |  | 11 (nRNP) | |  |  |
| NOR-90, % | 8 | 0 | N/A | 10 |  |  |  |  |  |  |  |  |  |  |  |  |  |  |  |  |  |  |  |
| Others, % |  |  |  |  |  |  |  |  |  |  |  |  | 0 (PM-Scl) |  |  | 33 (PM-1) |  |  |  |  |  |  | RF 29%, CCP 13%, APLA 29%, Mpo-anca 14% |

Abbreviations: ACA – anti-centromere antibody; APLA – anti-phospholipid antibodies; ARNAP – anti-RNA polymerase III; ATA – anti-topoisomerase I; CCP – anti-cyclic citrullinated antibody; Cen – centromeric pattern; CIE – counterimmunoelectrophoresis; CTD – connective tissue diseases; Cyto – cytoplasmic; H – homogeneous; H/Sp – homogeneous and speckled; LIA – line immunoassay; N/A – not available; NOR – nucleolar organizing region; RF – rheumatoid factor; MPO-ANCA – myeloperoxidase-antineutrophil cytoplasmic antibody.

Supplementary Table 4. Definitions of single-specificity among the studies summarized in Table 6

| Technique | Study | Total study population | Subpopulation positive for anti-Ku | Subpopulation positive for single-specificity anti-Ku | Definition of single-specificity based on exclusivity of these autoantibodies |
| --- | --- | --- | --- | --- | --- |
| Line immunoassay | Current study (2016) | 2140 | 24 (1.1%) | 13 (0.6%) | ACA, ATA, ARNAP, fibrillarin, NOR90, Th/To, Ro52, PDGFR, PM75, PM100 |
|  | Patterson [20], 2015 | 505 | 14 (2.8%) | 3 (0.6%) | ACA, ATA, ARNAP, fibrillarin, NOR90, Th/To, Ro52, PDGFR, PM75, PM100 |
|  | Graf [16], 2012 | 129 | 6 (5%) | 2 (1.6%) | ACA, ATA, ARNAP, fibrillarin, Th/To, PMScl, U1RNP |
|  | Villalta [24], 2012 | 210 | 10 (4.7%) | 6 (2.9%) | ACA, ATA, ARNAP, fibrillarin, NOR90, Th/To, Ro52, PDGFR, PM75, PM100 |
| Counterimmuno-electrophoresis | Cavazzana [14], 2013 | 560 | 13 (2.3%) | 10 (1.8%) | ENA (by CIE) (including at least anti-Ro, La, Ki, Jo1, PMScl, ACA, ATA) |
|  | Rozman [22], 2008 | 625 | 14 (2.2%) | 10 (1.6%) | Anti-Sm, U1RNP, Ro, La, ATA, Jo1, PMScl, SL, PCNA |
| Counterimmuno-electrophoresis + Immunoblot | Cavazzana [13], 2008 | 379 | 8 (2.1%) | 6 (1.6%) | ENA (by CIE) (including at least anti-Ro, La, Ki) |
|  | Francescini [32], 2002 | N/A | 7 (CIE), 6 (IB) | 4 | ENA (by CIE) (including at least anti-Ro, La, SL, PMScl) |
| Double immunodiffusion | Kuwana [17], 1994 | 275 | 8 (2.9%) | 7 (2.5%) | ACA, ATA, ARNAP, fibrillarin, ThRNP, PMScl, U1RNP |
|  | Mimori [19], 1981 | 66 | 8 (12%) | 0 | RNP, PM-1, SSA, other unidentified precipitin systems |
| Western blot | Rodriguez-Reyna [21], 2011 | 139 | 14 (10.1%) | N/A | N/A |
| Immunoblot | Yaneva [25], 1989 | 56 | 9 (16.1%) | 5 (9.1%) | Ro, La, Sm, nRNP, ATA, ACA |
| Dot blot | Rigolet [42], 2012 | N/A | 7 | 4 | ACA, ATA, Ro52, SSA-SSB, RNP, DNA, Sm, Jo1, PL7, PL12, Mi2, SRP, PMScl. Other overlapping antibodies that are not excluded from single-specificity: RF (2/7), anti-CCP (1/7), APLA (2/7), anti-thyroid (3/7), MPO-ANCA (1/7). |
| Immunoprecipitation | Kaji [48], 2014 | N/A | N/A | 44 | "SSc-related antibodies" not further specified; all tested for ACA, ATA, ARNAP, fibrillarin, Th/To, U1RNP, U2RNP, U5RNP, U4/6RNP, U11/12RNP, PMScl, synthetases, SRP, Mi2, MDA5, Tify1, NXP2, SAE, SSA (incl. Ro52), SSB, Sm, ribosomal |

Abbreviations: ACA – anti-centromere antibody; anti-CCP – anti-cyclic citrullinated peptide antibody; APLA – anti-phospholipid antibodies; ATA – anti-topoisomerase I antibody; ARNAP – anti-RNA polymerase III antibody; CIE – counterimmunoelectrophoresis; ENA – autoantibodies to extractable nuclear antigens; IB – immunoblot; MDA-5 – melanoma differentiation-associated protein 5 (also known as CADM-140); MPO-ANCA – myeloperoxidase anti-neutrophil cytoplasmic antibody; N/A – not available; NOR – nucleolar organizing region; PDGFR – platelet derived growth factor receptor; RF – rheumatoid factor; RNP – ribonucleoprotein; SRP – signal recognition particle; Tifγ1 – transcriptional intermediary factor-1.
